# Two Acute Stroke Patients Whose Lower Back and Lower Limb Pain Hampered Their Rehabilitation: The Effectiveness of Peripheral Nerve Blocks

**DOI:** 10.7759/cureus.68114

**Published:** 2024-08-29

**Authors:** Hiroyuki Dan, Kyongsong Kim, Tadahiro Ishiwada, Masaru Aoyagi, Yasuo Murai

**Affiliations:** 1 Department of Neurological Surgery, Shioda Memorial Hospital, Chiba, JPN; 2 Department of Neurological Surgery, Chiba Hokusoh Hospital, Chiba, JPN; 3 Department of Neurological Surgery, Nippon Medical School, Tokyo, JPN

**Keywords:** stroke, rehabilitation, lower limb pain, low back pain, entrapment neuropathy, endovascular treatment

## Abstract

Lower back and lower limb pain can hamper the rehabilitation of cerebral stroke patients. We report that peripheral nerve blocks enabled two patients to continue rehabilitation. Case 1 was an 83-year-old female with left hemiparesis due to cerebral infarction of the right basal ganglia. Rehabilitation started on the day after the stroke onset. On the 7^th^ post-stroke day, she reported right buttock and dorsal thigh pain. Lumbar MRI demonstrated no lumbar spinal canal stenosis and no nerve impingement. The middle cluneal nerve block alleviated her buttock pain. On the 29^th^ post-stroke day, she suffered severe pain on the medial side of the right knee. Blocking the infrapatellar branch of the saphenous nerve lessened that pain, she was able to walk without assistance, and rehabilitation was resumed. Case 2 was an 87-year-old female with sudden-onset left hemiparesis due to cardiogenic cerebral infarction. Intravenous thrombolysis and mechanical thrombectomy were performed. She presented with left hemiparesis due to infarcts at the right basal ganglia and the right temporal and parietal lobes. Her chronic low back pain worsened after admission and walking was difficult. Bilateral superior and middle cluneal nerve blocks improved her right lower back pain. Left low back pain was alleviated by sacroiliac joint blockage and rehabilitation was possible due to the absence of back pain. The strain on the lower back and lower limbs attributable to paresis due to stroke may lead to entrapment neuropathy. Peripheral nerve blockage is relatively simple and safe and may be useful in acute stroke patients whose rehabilitation is difficult due to pain.

## Introduction

Back and lower limb pain can be an obstacle to post-stroke rehabilitation and reduce the patient’s activities of daily living (ADL) [1]. These pains can appear due to not only orthopedic diseases but also entrapment neuropathy [2-6]. MRI and X-ray studies are not always diagnostic in patients with entrapment neuropathy of the lower back and lower limbs. Acquisition of a Tinel sign at the entrapment site and symptom improvement after nerve blocks help to diagnose some patients at the bedside [1,6-8]. We encountered two patients in whom peripheral nerve blocks reduced their lower back and lower limb pain that hindered rehabilitation after an acute stroke. There are few reports on compressive peripheral neuropathy as a cause of lumbar and lower limb pain after a stroke. We report that focusing on entrapment peripheral neuropathy as a cause of lumbar and lower limb pain following a stroke was useful in improving symptoms.

## Case presentation

Case 1

**Figure 1:**
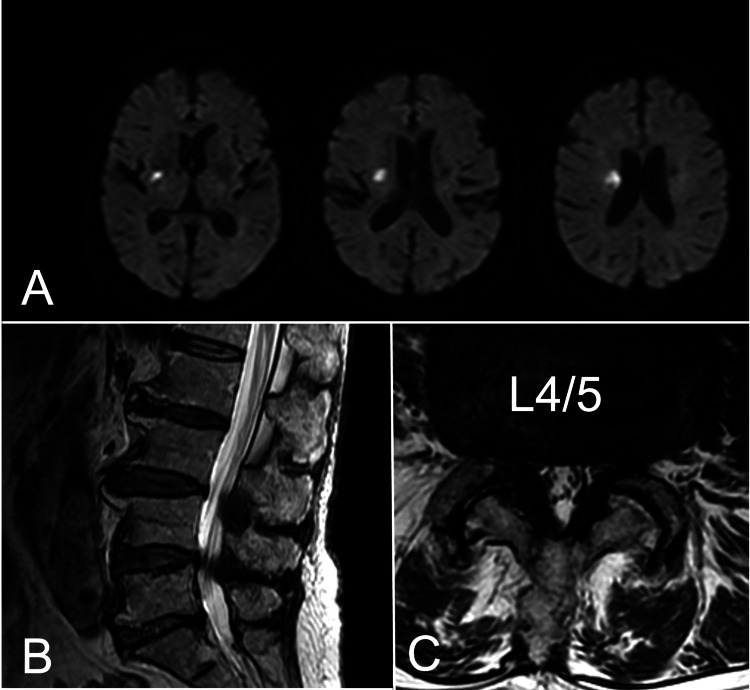
Initial head MRI and lumbar spine MRI after admission. (A) Diffusion-weighted brain MRI (axial images) performed on the day of admission. Note the small cerebral infarct in the right corona radiata. (B, C) T2-weighted lumbar spine MRI (B: sagittal image; C: axial image) showing lumbar spinal canal stenosis at L4/5.

An 83-year-old female was able to pursue her ADL and had no relevant past history. Upon awaking, she experienced sudden-onset left hemiparesis and dysarthria. A brain MRI at a local hospital revealed cerebral infarction. At the time of admission to our hospital, her consciousness level was 1 on the Japan Coma Scale (JCS) and she presented with dysarthria and left hemiparesis (grade 4 on the Manual Muscle Testing (MMT)). Brain MRI showed cerebral infarction in the right basal ganglia without major cerebral artery stenosis or occlusion (Figure 1A). Rehabilitation started on the 1st day after onset and her neurological symptoms did not worsen. She was treated with argatroban (60 mg/day for the first two days and 10 mg/day for the subsequent five days) and edaravone (60 mg/day) for seven days, with oral aspirin (100 mg) and clopidogrel (75 mg) for two weeks, and then with aspirin (100 mg).

On the 7th post-onset day, she reported right buttock and dorsal thigh pain elicited by walking. The numerical rating scale (NRS) score for pain was 7 and her walking distance was reduced from 50 meters to 7 meters. Lumbar MRI demonstrated spondylotic changes due to aging and there was moderate to severe spinal lumbar stenosis at L4/5 (Figures 1B, 1C). Her buttock pain was located in the middle cluneal nerve area, and a Tinel sign was confirmed between the posterior superior and inferior iliac spine, suggesting that the pain was due to middle cluneal nerve entrapment. This pain was not present before hospitalization but appeared after the patient developed a cerebral infarction and began rehabilitation. Oral mirogabalin was not effective. The right middle cluneal nerve block (2 ml of 1% lidocaine) delivered on the 19th post-onset day improved her buttock and thigh pain (NRS 3) and on the 24th day, she was able to walk two rounds of 10 meters with a break in between. On the 29th day, she reported severe pain on the medial side of the right knee. The site was on the anterior medial side of the knee joint, the entrapment point of the infrapatellar branch of the saphenous nerve. Blocking with 3 ml of 1% lidocaine reduced the NRS from 6 to 1. She was able to walk 20 meters with the aid of a cane and post-stroke rehabilitation was resumed. She was transferred to a rehabilitation hospital on the 33rd post-stroke day. Her modified Rankin scale (mRS) score was 4 and she was discharged four months after the stroke with an mRS of 1. At the final visit six months after the stroke, she was able to walk unassisted with some lower back and knee pain.

Case 2

**Figure 2:**
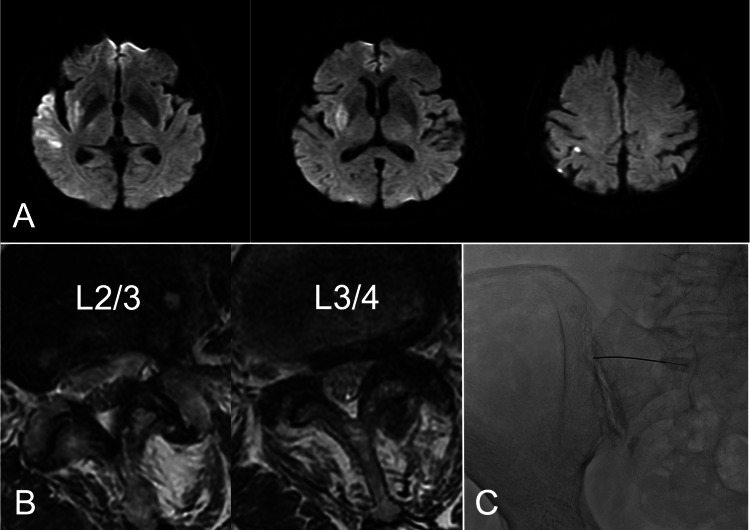
Initial head MRI, lumbar spine MRI after admission, and left sacroiliac joint fluoroscopy image. (A) Diffusion-weighted brain MRI (axial images) obtained two days after admission. Scattered cerebral infarcts in the right temporal lobe, the parietal lobe, and the basal ganglia are shown. (B) T2-weighted lumbar spine MRI (axial images) showing lumbar spinal canal stenosis at L2/3 and L3/4. (C) Left sacroiliac joint blockage under fluoroscopy.

An 87-year-old female with a history of ovarian cyst surgery was being treated for hypertension and dyslipidemia. She had been in control of her ADL despite experiencing intermittent claudication due to chronic lower back pain. She developed sudden-onset left hemiparesis and was admitted to our hospital. Her consciousness was JCS grade 3; her left hemiparesis was 2/5 according to the MMT and there was a right conjugate deviation. Diffusion-weighted brain MRI revealed a faint high signal in the right temporal lobe and insular cortex. Fluid-attenuated inversion recovery MRI detected no high signal in that area. Brain magnetic resonance angiography confirmed occlusion of the inferior trunk of the right middle cerebral artery. After intravenous thrombolysis and mechanical thrombectomy, her MMT score for left paresis was 4/5. A brain MRI scan obtained two days post admission showed infarcts at the right basal ganglia and the right temporal and parietal lobes (Figure 2A). Edoxaban (30mg/day) was started to prevent further cerebral infarction, and rehabilitation was started.

However, her chronic lower back pain worsened, and she had difficulty walking a distance of 5 meters to the toilet. Lumbar MRI confirmed spinal canal stenosis at L2/3 to L3/4 (Figure 2B) and a Tinel sign was present in the left and right superior and middle cluneal nerve regions, suggesting concomitant peripheral entrapment neuropathy. As her pain did not improve, we delivered bilateral superior and middle cluneal nerve blocks on the 13th day after admission (2 ml of 1% lidocaine each). Her right-side lower back pain improved from NRS 8 to 0, and her left-side pain from NRS 8 to only grade 6. Examination of the residual pain showed that the left sacroiliac joint score was 6 of 9 (one finger test: 3 points; pain when sitting in a chair: 1 point; tenderness at the posterior superior iliac spine: 1 point; tenderness at the sacrotuberous ligament: 1 point); it was suspected that sacroiliac joint disorder was causing her lower back pain. On the 16th post-stroke day, we blocked four sites at the sacroiliac joint (2 ml of 1% lidocaine in four locations) under fluoroscopy. This lowered the NRS grade to 1 (Figure 2C) and she was able to walk more than 50 meters without assistance.

On the 23rd post-stroke day after she was discharged, her mRS was grade 2. Three months after the stroke, she experienced a recurrence of low back pain, but she was able to perform ADL as she had prior to her hospitalization. She remains under observation on an outpatient basis.

## Discussion

The symptoms of peripheral nerve entrapment affecting the lower back and lower limbs are easily misinterpreted because they are similar to the symptoms of lumbar spine disease and they can persist after lumbar spine surgery [2-9]. We encountered two patients whose rehabilitation after a cerebral stroke was hampered by lower back and lower limb pain. Successful nerve blockage to treat overlooked peripheral nerve entrapment eliciting the pain facilitated the continuation of their post-stroke rehabilitation.

The superior and middle cluneal nerves are pure sensory nerves and their entrapment around the iliac crest has been shown to account for 10% of all low back pain [6-8,10-13]. Besides low back pain, patients may report concomitant lower limb symptoms and intermittent claudication. Consequently, lumbar spinal canal stenosis must be differentiated from nerve entrapment although both may be present at the same time [8-12]. In both of our patients, block therapy for cluneal neuropathy effectively addressed their low back pain that hampered rehabilitation during the acute phase of stroke.

The pathogenesis of cluneal neuropathy remains unclear. It may be concomitant with lumbar spine diseases and it may be related to increased muscle tone in the lumbar area and the buttocks and abnormal posture [3,7,8,10-12]. As lumbar MRI scans revealed degenerative changes in the lumbar spine of both elderly patients, we cannot assert that stroke-related paralysis was responsible for the lower back pain. In case 1, low back pain on the non-paralyzed side developed after the start of post-stroke rehabilitation, suggesting that muscle activity characteristic of hemiplegic patients was associated with cluneal neuropathy [14,15]. Sato showed muscle activity in hemiplegic patients and suggested that excessive use of the healthy muscles, increased muscle tone on the healthy side, trunk rotation, and overactivity of the erector spinae muscles could be contributing factors to cluneal neuropathy [7,15]. A similar mechanism was considered in case 2.

**Figure 3:**
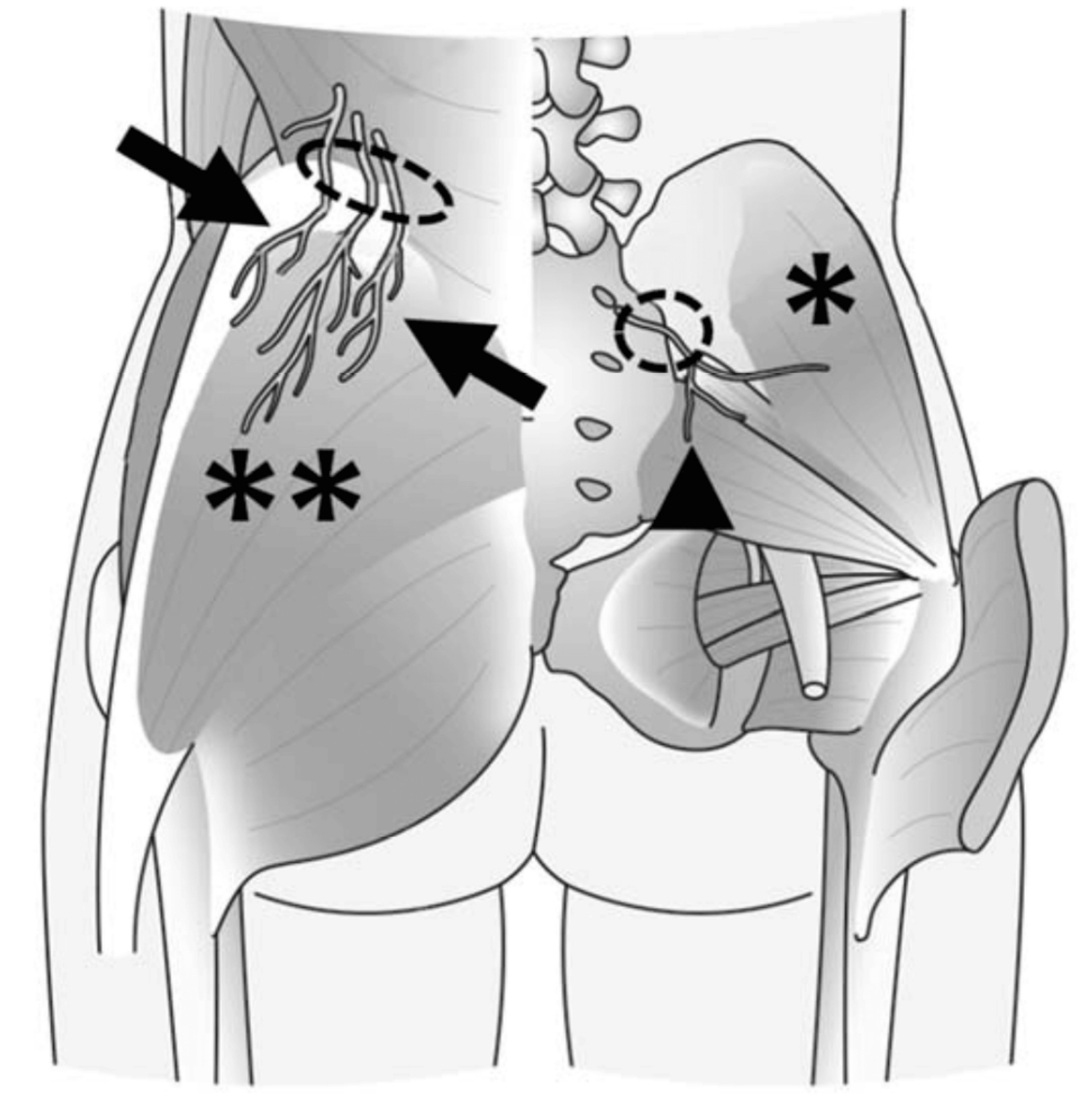
Superior and middle cluneal nerves and the entrapment site. Superior cluneal nerve (arrows) and middle cluneal nerve (arrowhead). Dotted circles indicate the sites of the Tinel sign and the nerve blocks. * Gluteus medius muscle. ** Gluteus maximus muscle. This figure has been adapted from Isu et al. [7], which is an open-source article distributed under the terms and conditions of the Creative Commons Attribution Non-Commercial License (http://creativecommons.org/licenses/by-nc/4.0/).

When low back pain is due to cluneal nerve entrapment, a Tinel sign is elicited on the iliac crest at the site of nerve compression (Figure 3). A diagnosis is made when pain is relieved by superior and middle cluneal nerve blocks at the compression sites [6,7,10,13]. Specific treatments include superior and middle cluneal nerve blocks and surgical neurolysis. In 28-100% of patients with superior cluneal nerve entrapment, blocks are effective [3,7,9,13].

The sacroiliac joint is rigid and reinforced by strong ligaments. Low back pain attributable to sacroiliac joint pain has been reported in approximately 30% of low back pain patients [16]. It is characterized by pain along the sacroiliac joint, is centered on the posterior superior iliac spine, and the pain tends to worsen when sitting. The sacroiliac joint score is useful for distinguishing between sacroiliac joint pain and lumbar disease (Table 1). When the total score is 4 or higher, low back pain originating at the sacroiliac joint is suspected. A 70% or greater pain reduction by sacroiliac joint blockage is diagnostic [14,17]. Causes of low back pain include sacroiliac joint disorders and entrapment of the superior and middle cluneal nerves, which need to be differentiated. When a simple middle cluneal nerve block is not effective, a diagnostic sacroiliac joint block should be considered [8,11]. In case 2, hemiplegia after the stroke may have exacerbated the shear force on the sacroiliac joint, exacerbating the sacroiliac joint disorder [18]. Persistent microscopic incompatibility caused by shear force on the sacroiliac joint leads to inflammation and aggravates pain [18,19].

**Table 1:** Scoring system for sacroiliac joint pain. PSIS: posterosuperior iliac spine; STL: sacrotuberous ligament.

Item	Score
1. One-finger test	3
2. Groin pain	2
3. Pain while sitting on a chair	1
4. Sacroiliac joint shear test	1
5. Tenderness of PSIS	1
6. Tenderness of STL	1
Total	9

**Figure 4:**
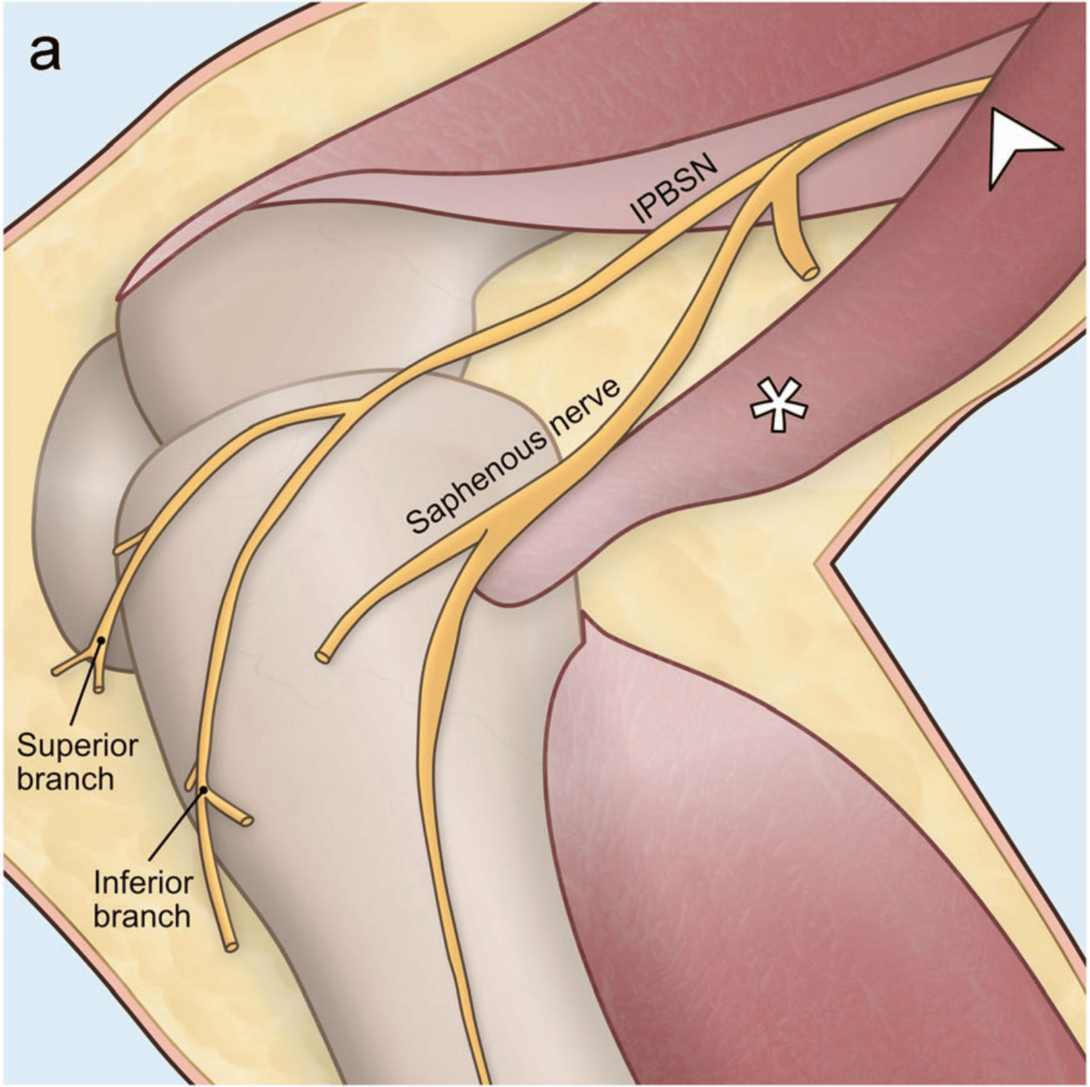
The course of the infrapatellar branch of the saphenous nerve (IPBSN). The infrapatellar branch of the saphenous nerve divides into superior and inferior branches, providing sensation to the skin at the front of the knee, the anterolateral area of the upper leg, and the anterior inferior joint capsule. It originates within the subsartorial canal (arrowhead) piercing the fascia anterior or through the sartorius muscle (asterisk). This figure has been adapted from Yang et al. [20], which is an open-source article distributed under the terms and conditions of the CC BY license.

The infrapatellar branch of the saphenous nerve is also a pure sensory nerve. It innervates the anterior medial skin of the knee and the anterior lateral and anterior inferior joint capsule of the proximal lower leg (Figure 4) [20]. The nerve site can be damaged during total knee arthroplasty; saphenous nerve entrapment by the sartorius muscle has been reported [20]. In case 1, knee pain elicited by the infrapatellar branch of the saphenous nerve on the non-paralyzed side was developed on the 29th post-stroke day. Hemiplegic patients tend to overuse the muscles on their healthy side to increase stability, which may have caused the healthy lower limb muscles to become overtense and compress the saphenous nerve. The usefulness of diagnostic nerve blocks and subsequent long-term pain alleviation, as observed in case 1, have been reported [6,20]. Post-stroke occurrence of lumbar and lower limb pain due to orthopedic conditions such as spinal stenosis and disc herniation has been reported and is often encountered [2,14]. However, there are few reports addressing compressive peripheral neuropathy in the lumbar and lower limbs. In these cases, focusing on and addressing compressive peripheral neuropathy as a cause of lumbar and lower limb pain that developed or worsened after a stroke proved to be effective.

## Conclusions

In patients with acute cerebral stroke, peripheral nerve blockage may effectively address lower back and lower limb pain that hinders rehabilitation. The strain on the lower back and lower limbs due to stroke-related paresis may result not only in orthopedic diseases but also in entrapment neuropathy. The delivery of peripheral nerve blocks at the bedside is relatively simple and safe and should be considered in patients whose post-stroke rehabilitation is hampered by lower back and lower leg pain.
